# Selective detection of cyanogen halides by BN nanocluster: a DFT study

**DOI:** 10.1007/s00894-017-3312-1

**Published:** 2017-03-29

**Authors:** E. Vessally, F. Behmagham, B. Massuomi, A. Hosseinian, K. Nejati

**Affiliations:** 1 Department of Chemistry, Payame Noor University, Tehran, Iran; 2 Department of Engineering Science, College of Engineering, University of Tehran, PO Box 11365-4563, Tehran, Iran

**Keywords:** Electronic properties, Gas sensor, Nanostructure, Boron nitride, DFT

## Abstract

The electronic sensitivity and adsorption behavior toward cyanogen halides (X–CN; X = F, Cl, and Br) of a B_12_N_12_ nanocluster were investigated by means of density functional theory calculations. The X-head of these molecules was predicted to interact weakly with the BN cluster because of the positive σ-hole on the electronic potential surface of halogens. The X–CN molecules interact somewhat strongly with the boron atoms of the cluster via the N-head, which is accompanied by a large charge transfer from the X–CN to the cluster. The change in enthalpy upon the adsorption process (at room temperature and 1 atm) is about −19.2, −23.4, and −30.5 kJ mol^−1^ for X = F, Cl, and Br, respectively. The LUMO level of the BN cluster is largely stabilized after the adsorption process, and the HOMO–LUMO gap is significantly decreased. Thus, the electrical conductivity of the cluster is increased, and an electrical signal is generated that can help to detect these molecules. By increasing the atomic number of X, the signal will increase, which makes the sensor selective for cyanogen halides. Also, it was indicated that the B_12_N_12_ nanocluster benefits from a short recovery time as a sensor.

## Introduction

Cyanogen halides (X–CN, X = halogen) are colorless, chemically reactive, lachrymatory (tear-producing), and volatile compounds with a linear structure [1]. They are highly poisonous agents, and symptoms of exposure may include paralysis, vomiting, drowsiness, coughing, convulsion, throat confusion, edema, and death [1, 2]. Thus, finding a portable, fast response, highly sensitive, simple, and reliable sensor for X–CN detection is of great importance. Previous methods suggested and investigated include spectrophotometric, electrochemical, and gas chromatographic approaches [3–5]. Most of these procedures need complicated instruments and are expensive. With the advent of nanotechnology, gas sensor development has accelerated due to the high adsorption capacity, high surface/volume ratio and unique electronic sensitivity of nanostructures [6, 7]. To date, numerous nanostructured material based sensors have been introduced for different gases by both experimental researchers and theoreticians [8–14]. Boron nitride (BN) nanostructures are an important class of nanostructure with wide band gap, special electronic, optical and magnetic properties [15–18]. Many studies have focused on the fullerene-like BN nanoclusters, nanosheets and nanotubes as gas sensors [19–24].

The stability and geometries of (BN)_*n*_ (*n* = 4–30) nanoclusters have been explored previously by different groups [25–27]. It has been indicated that the B_12_N_12_ nanocluster has a magic structure and is highly stable; this nanocluster has also been successfully synthesized [25]. Several studies have focused on the potential use of the B_12_N_12_ nanocluster in hydrogen storage, Li-ion batteries, drug delivery, and gas sensors [26–33]. Very recently, it was demonstrated that a fluoride-encapsulated B_12_N_12_ nanocluster is a promising candidate for anode materials in Li-ion batteries [29]. The hydrogen storage capability of this nanocluster was explored by Jia et al. [33] using ab initio molecular orbital theory. It has also been revealed that B_12_N_12_ is the most stable nanocluster among different X_12_Y_12_ (X = Al or B and Y = N or P) nanoclusters [34]. Herein, we investigate the interaction between different X–CN (X = F, Cl, and Br atoms) molecules, and the B_12_N_12_ nanocluster using density functional theory (DFT) calculations to explore the potential application of B_12_N_12_ nanocluster as a chemical sensor.

## Computational methods

Natural bond orbitals (NBO), molecular electrostatic potential (MEP) and density of states (DOS) analyses, geometry optimizations, and energy predictions were performed on a B_12_N_12_ nanocluster and different X-CN/B_12_N_12_ complexes at B3LYP level of theory with 6-31G (d) basis set as implemented in the GAMESS suite of programs [35]. The B3LYP functional was augmented with an empirical dispersion term [36] (B3LYP-D) to improve its reliability in prediction of noncovalent interactions. The B3LYP has been demonstrated to be a commonly employed density functional in the investigation of different nanomaterials [37–53]. In addition, it has been specified to deliver a well-organized and robust basis for III–V semiconductor calculations [54]. The GaussSum program [55] was selected to obtain DOS plots. Vibrational frequency calculations were performed to verify that all the geometries are true minima with positive Hessian eigenvalues. Adsorption energy was calculated as follows

1 $$ {\mathrm{E}}_{\mathrm{ad}} = {\mathrm{E}}_{\mathrm{tot}}\left(\mathrm{X}-\mathrm{CN}/{\mathrm{B}}_{12}{\mathrm{N}}_{12}\right)-{\mathrm{E}}_{\mathrm{tot}}\left({\mathrm{B}}_{12}{\mathrm{N}}_{12}\right)-{\mathrm{E}}_{\mathrm{tot}}\left(\mathrm{X}-\mathrm{CN}\right) $$

where* E*_tot_(X–CN/ B_12_N_12_) is total energy of X–CN/ B_12_N_12_ complex and* E*_tot_(B_12_N_12_) and* E*_tot_(X–CN) are total energies of isolated B_12_N_12_ cage, and X–CN molecules, respectively.

The enthalpy change (Δ*H*_ad_) of X–CN adsorption at room temperature and 1 atm pressure was calculated as follows:

2 $$ \varDelta {\mathrm{H}}_{\mathrm{ad}}=\mathrm{H}\left(\mathrm{X}-\mathrm{CN}/{\mathrm{B}}_{12}{\mathrm{N}}_{12}\right)-\mathrm{H}\left({\mathrm{B}}_{12}{\mathrm{N}}_{12}\right)\hbox{--} \mathrm{H}\left(\mathrm{X}-\mathrm{CN}\right) $$

where* H*(X-CN/ B_12_N_12_) is the enthalpy of the complex, and* H*(B_12_N_12_) and H(X–CN) are the enthalpies of the pristine B_12_N_12_ and X–CN molecule, respectively. Zero-point energy and basis set superposition error (BSSE) corrections [56] were included in the Δ*H*_ad_ and adsorption energy calculations. Assessing the sensitivity of the sensor, the shift of the HOMO–LUMO energy gap (*E*_g_) was computed by:

3 $$ \varDelta {\mathrm{E}}_{\mathrm{g}}=\left[\left({\mathrm{E}}_{\mathrm{g}2} - {\mathrm{E}}_{\mathrm{g}1}\right)/{\mathrm{E}}_{\mathrm{g}1}\right]*100\ \% $$

where* E*_g1_ and* E*_g2_ are the values of the* E*_g_ for bare B_12_N_12_ and the X–CN adsorbed state, respectively.

## Results and discussion

### Specifications of B_12_N_12_ nanocluster

**Fig. 1:**
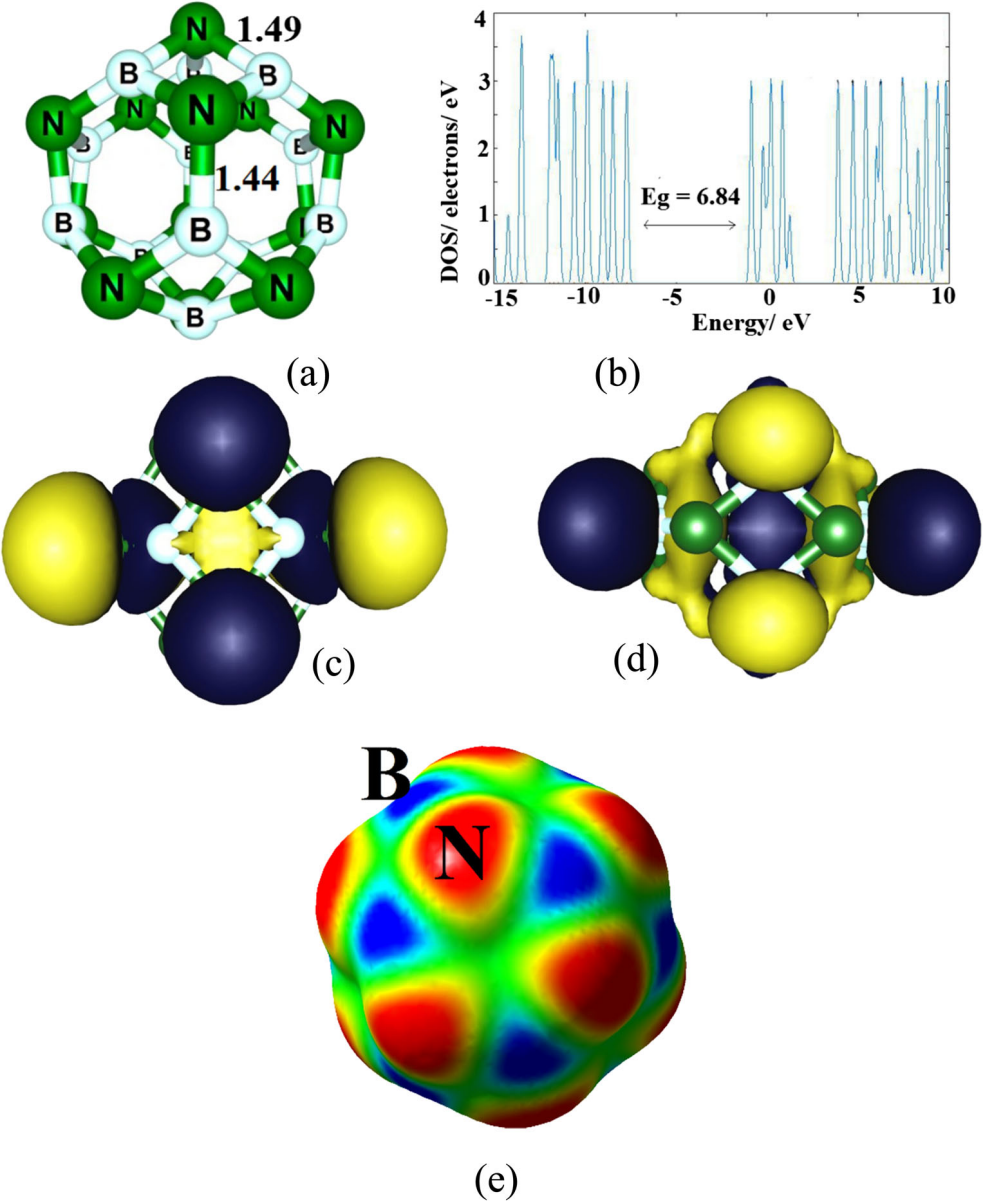
**a** Optimized structure, **b** density of states, **c** HOMO, and **d** LUMO profiles of the pristine B_12_N_12_ nanocluster. Distances in Å. The* E*_g_ indicates the HOMO–LUMO energy gap. **e** Calculated electrostatic potential on the molecular surface of B_12_N_12_. Color ranges, in kcal mol^−1^:* red* greater than 15,* yellow* between 15 and 5,* green* between 5 and −5,* blue* less than −5 (negative)

As shown in Fig. 1, the B_12_N_12_ nanocluster is made of eight hexagons and six tetragons with *T*_h_ symmetry. Structurally, two individual B–N bonds are distinguished, one of which is shared by two hexagons (66-bond) and another between a tetragon and a hexagon (46-bond) with average bond lengths of 1.44 Å and 1.49 Å, respectively, in good agreement with the experimental results [25]. The 46-bond is larger than the 66-bond due to the higher strain on the tetragonal ring. The range of calculated vibrational frequencies is from 323^−1^ to 1446 cm^−1^, representing that the geometry is a true stationary point on the potential energy surface. The DOS plot indicates that it is a wide gap (∼6.84 eV) nanocluster in which the HOMO and LUMO are located mainly on the N and B atoms, respectively (Fig. 1). Figure 1 also shows the MEP on the surface of the BN nanocluster. It can be seen that the negative regions above the nitrogen atoms are stronger than the positive ones of the boron atoms; the former have local maxima of −16 to −18 kcal mol^−1^, while the local minima of the latter are only +5 to +7 kcal mol^−1^. This may be due to the large curvature and lone pairs of the N atoms.

### Adsorption of X–CN molecules on B_12_N_12_

**Fig. 2:**
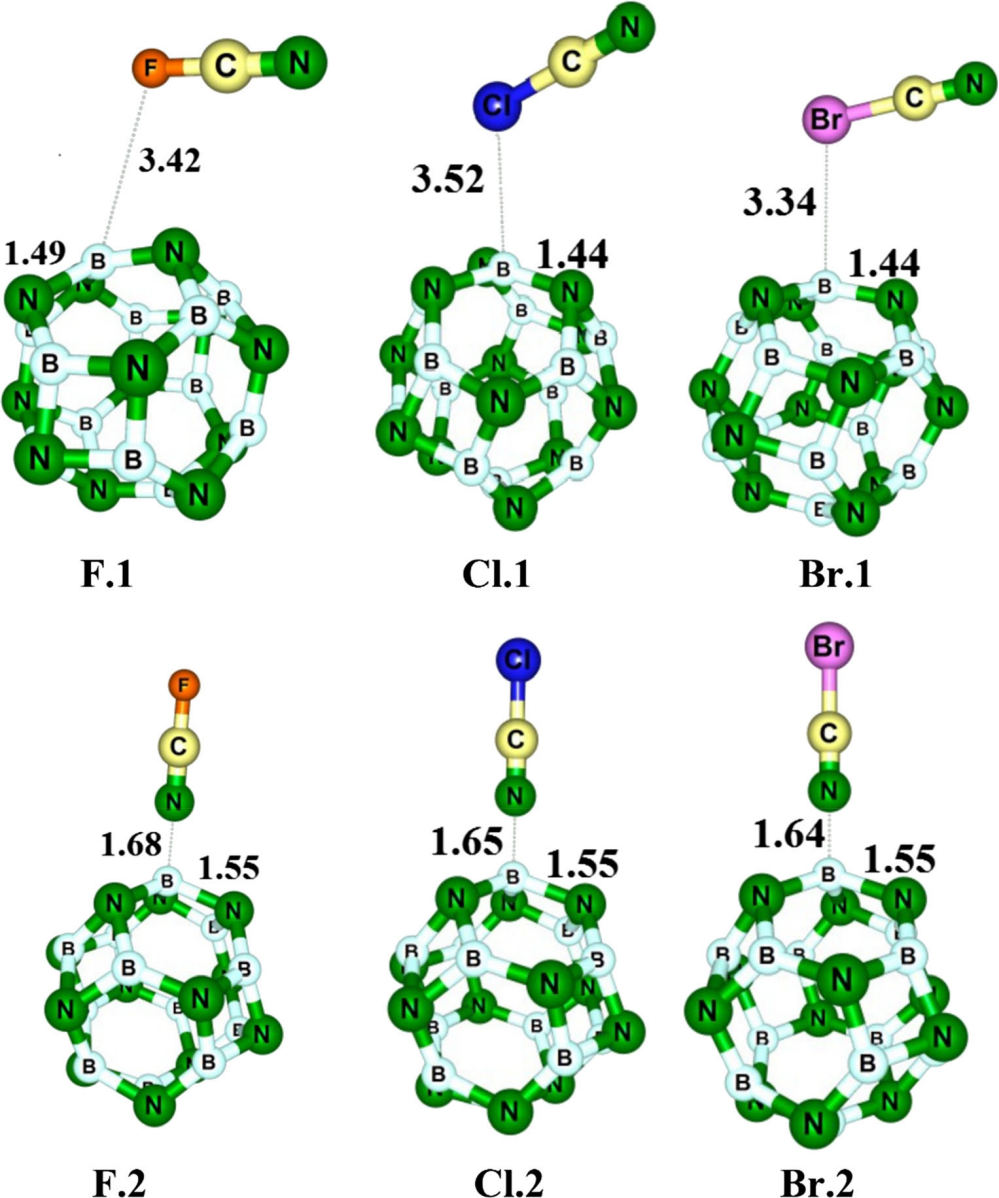
Optimized structures of X–CN/B_12_N_12_ complexes. Distances in Å

For X–CN molecules, the chemistry of molecules indicates that nucleophile heads X or N should attack the electrophile sites (B atoms) of the BN cluster. Thus, we optimized the initial structures in which the X or N atom of the molecules are located on a B atom of the cage and then a relaxation occurred. Also, in another attempt, we located the X–CN molecule on a hexagonal ring so that both the X and N heads wer close to to the B sites. Finally, we found two local minima for each molecule as shown in Fig. 2. When the molecules were located on the hexagonal ring, they are reoriented to the structures in which the molecule is attached from its N head to the B atom of the cluster.

**Fig. 3:**
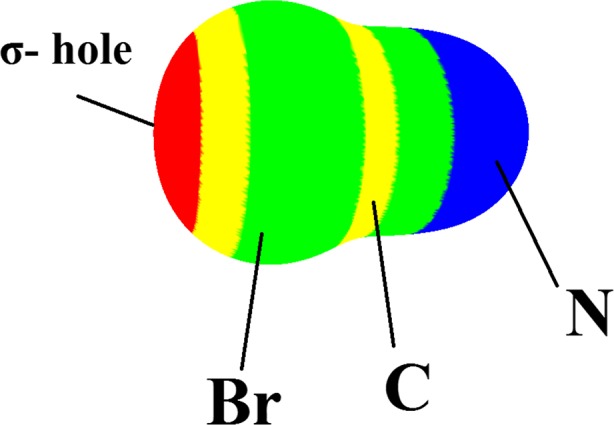
Molecular surface electrostatic potential (MEP) of Br–CN, computed on the 0.001 au contour of the electronic density. Color ranges, in kcal mol^−1^, are:* red* greater than 15,* yellow* between 15 and 5,* green* between 5 and −5,* blue* less than −5 (negative). The σ-hole along the extension of the Br–C bond is shown in* red*

Table 1 shows that the complexes in which the molecule is attached from its N head to the B atom of the cluster are more stable than those in which it is attached from the X head. For example, the adsorption energy of complex **F.2** (Fig. 2) in which the F–CN is linked from the N atom to the B atom of the cluster is about −24.2 kJ mol^−1^ and the Δ*H*_ad_ is about −19.2 kJ mol^−1^, while the adsorption energy and Δ*H*_ad_ are about −8.7 kJ mol^−1^ and −6.6 kJ mol^−1^ for complex **F.1** in which the F–CN is attached from the F head to the B atom. The weak interaction of halogens with B sites is somewhat enigmatic: the halogens are viewed as usually being negative in nature; why should not they interact strongly with electron deficient sites? This matter can be understood based on the σ-hole concept [57]. The σ-holes are regions of positive electrostatic potential of halogens along the extensions of the covalent bonds, which were initially introduced by Murray et al. [58]. As shown in Fig. 1, the B atoms have a positive electrostatic potential, which hinders adsorption of X–CN from its X-head. For example, Fig. 3 illustrates the σ-hole on the Br–CN as a representative model. The positive electrostatic of the σ-hole in halogens somewhat precludes a strong interaction between the halogen and the positive electrostatic surface of the B atoms. The molecular surface electrostatic potential (MEP) has been frequently used as a guide to reactive behavior [59–62].

**Table 1:** Adsorption energy (*E*_ad_, kJ mol^−1^), change of enthalpy (∆H_ad_ kJ mol^−1^) for different cyanogen halides (X–CN; X = F, Cl, and Br) adsorption on the B_12_N_12_ nanocages. Vibrational frequencies and bond lengths of C–N and C–X bonds of cyanogen halides in different complexes. The numbers in parentheses are values for the free molecule. Complexes are shown in Fig. 2

Complex	*E*_ad_	∆H_ad_	υ_C–N_ (cm^−1^)	υ_C–X_ (cm^−1^)	R_C–N_ (Å)	R_C–X_ (Å)
**F.1**	−8.7	−6.6	2427 (2429)	1094 (1098)	1.161 (1.161)	1.273 (1.273)
**Cl.1**	−9.2	−7.5	2326 (2337)	741 (742)	1.163 (1.163)	1.646 (1.646)
**Br.1**	−10.8	−8.7	2309 (2309)	578 (580)	1.163 (1.163)	1.793 (1.793)
**F.2**	−24.2	−19.2	2547 (2429)	1173 (1098)	1.148 (1.161)	1.257 (1.273)
**Cl.2**	−29.6	−23.4	2410 (2337)	820 (742)	1.152 (1.163)	1.626 (1.646)
**Br.2**	−35.1	−30.5	2387 (2309)	678 (580)	1.152 (1.163)	1.772 (1.793)

By increasing the atomic number of the X atom, the interaction between the cyanogen and the cluster becomes stronger, which may be due to the fact that the larger molecules have larger polarizability, and thus show stronger interaction in the case in which the X–CN interacts with its X atom. But in cases where this molecule interacts with its N atom via the B site, the electron withdrawing nature of F, Cl and Br atoms may affect the interaction. Fluorine has the highest electronegativity, and, significantly, can withdraw electrons from the –CN group, compared to Cl and Br atoms. Thus, it can significantly weaken the interaction, as was shown in Table 1. The adsorption energy for **F.2**, **Cl.2** and **Br.2** is about −24.2, −29.6, and −35.1 kJ mol^−1^, respectively, indicating that the order of the reactivity of X–CN molecules toward BN cage is Br–CN > Cl–CN > F–CN.

When the adsorption process occurred from the X head, no discernable local structural deformation occurred and the molecules were located at a somewhat large distance from the cage, while upon the adsorption process via the N head, the adsorbing B atom is projected out slightly and the corresponding N–B–N angles decrease, indicating a stronger interaction. Table 1 lists the vibrational frequencies of X–C and C–N bonds of X–CN molecules in the free state and in complex forms; the corresponding bond lengths are also indicated. It can be seen that, in the X-head adsorption, neither the vibrational frequencies nor the bond lengths are changed markedly, indicating a noncovalent interaction. In the free X–CN molecules, the vibrational frequency of the C–N bond is about 2547 cm^−1^, 2410 cm^−1^, and 2387 cm^−1^ for X = F, Cl, and Br, respectively. This trend indicates that the stronger electron-withdrawing atom with higher electronegativity strengthens the C–N bond more and increases the bond order. This may be because of more electron-withdrawing from the antibonding orbital of the C–N bond.

After the adsorption process via the N-head, the vibrational frequency of the C–N bond is decreased significantly in the order Br > Cl > F. It seems that charge transfer from the molecule to the cluster may be responsible for the frequency reduction because of electron depletion from the antibonding orbital of the C–N bond. NBO analysis indicates that the charge transfer from X–CN is about 0.287 *e*, 0.311 *e*, and 0.324 *e* (Table 2) for X = F, Cl, and Br, respectively, which is in agreement with the trend of vibrational frequency reduction. Also, C–N bond length is somewhat shortened after the adsorption process, which is consistent with the charge transfer and frequency change. By electron reduction on the –CN group, its interaction with the high electron –X group becomes stronger, and the C–X bonds are shortened, as shown in Table 1, and their vibration frequencies are also increased.

**Table 2:** The energies of HOMO, LUMO, and HOMO−LUMO gap (*E*_g_) in eV for different structures. %∆*E*_g_ indicates the change in* E*_g_ after the adsorption process.* Q* is the calculated natural bond orbital (NBO) charge on the adsorbed X-CN (X = F, Cl, and Br) molecule. The complexes are shown in Fig. 2

Structure	*E*_HOMO_	*E*_LUMO_	*E*_g_	%∆*E*_g_	*Q*
B_12_N_12_	−7.70	−0.86	6.84	-	-
**F.1**	−7.78	−0.95	6.83	−0.2	0.001
**Cl.1**	−7.79	−0.98	6.81	−0.4	0.005
**Br.1**	−7.76	−1.15	6.61	−3.3	0.018
**F.2**	−6.87	−1.26	5.61	−17.9	0.287
**Cl.2**	−6.79	−1.87	4.92	−28.0	0.311
**Br.2**	−6.74	−2.49	4.25	−37.9	0.324

### Electronic properties

The main purpose this work was to explore the capability of B_12_N_12_ to detect X–CN gases. In addition to expensive experimental methods, numerous computational approaches have been used to investigate the sensing behavior of different nanostructures toward several poisonous gases [63–72]. One of the most widespread theoretical methods [11, 73–79] depends on the* E*_g_ change of the sensor upon gas adsorption. The conduction electron population is responsible for the electrical conductivity in a semiconductor, which can be formulated as [80]:

4 $$ \mathrm{N}=\mathrm{A}\ {\mathrm{T}}^{3/2} \exp \left(-{\mathrm{E}}_{\mathrm{g}}/2\mathrm{kT}\right) $$

where k is Boltzmann’s constant, and A is a constant with unit electrons/m^3^K^3/2^. A gas sensor operates based on the change of its electrical conductivity upon the gas adsorption and charge transfer. Equation 4 indicates that the population of conduction electrons of the B_12_N_12_ nanocluster will change exponentially by changing the* E*_g_ and will thus alter the electrical conductivity.

**Fig. 4:**
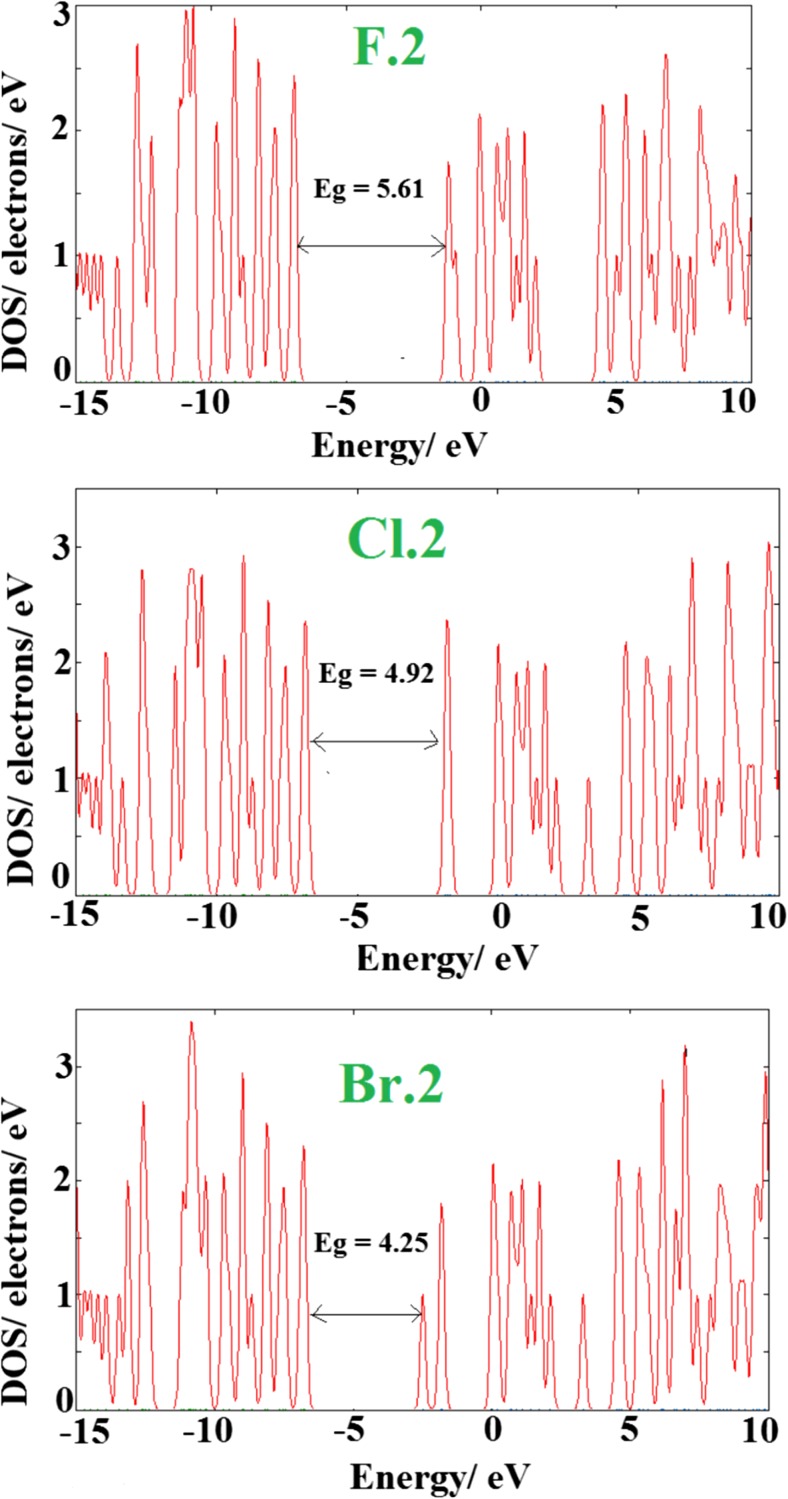
Density of states (DOS) plots of the different complexes shown in Fig. 2

Table 2 indicates that, upon the adsorption process via X-head, the HOMO, LUMO and* E*_g_ are not changed meaningfully, and also the NBO charge transfer is negligible, while the adsorption process from N-head significantly changes the electronic properties of the cluster, as shown by the DOS plots in Fig. 4. It should be noted that, in reality, the most favorable interaction will be from the N-head because of the large energy release. The DOS plots indicates that, after the adsorption process, new states appeared within the* E*_g_ that significantly reduce it. Overall, HOMO levels are destabilized slightly, and LUMO levels are largely stabilized, and thus* E*_g_ is decreased. NBO charge analysis demonstrated that, compared to the X-head adsorptions, in the case of N-head adsorption a large charge is transferred from the X–CN to the cluster, which may be responsible for the large electronic property changes accompanying the structural deformations.

By increasing the atomic number of the halogen in the X–CN molecule, the LUMO level is stabilized much more, and the charge transfer is also increased more. Thus, the* E*_g_ is decreased more, which increases exponentially the electrical conductivity. In the case of complex **Br.2**, after the adsorption of Br–CN, the LUMO level is shifted from −0.86 eV in the bare BN cage to −2.49 eV in this complex, indicating a large stabilization. The LUMO levels of complexes **F.2** and **Cl.2** are about −1.226 eV and −1.87 eV, respectively, which are less stabilized compared to that of the complex **Br.2**. Also, the* E*_g_ of the **Br.2** complex is reduced by about 37.9%, i.e., a reduction of about 17.9% and 28% for **F.2** and **Cl.2** complexes, respectively.

**Fig. 5:**
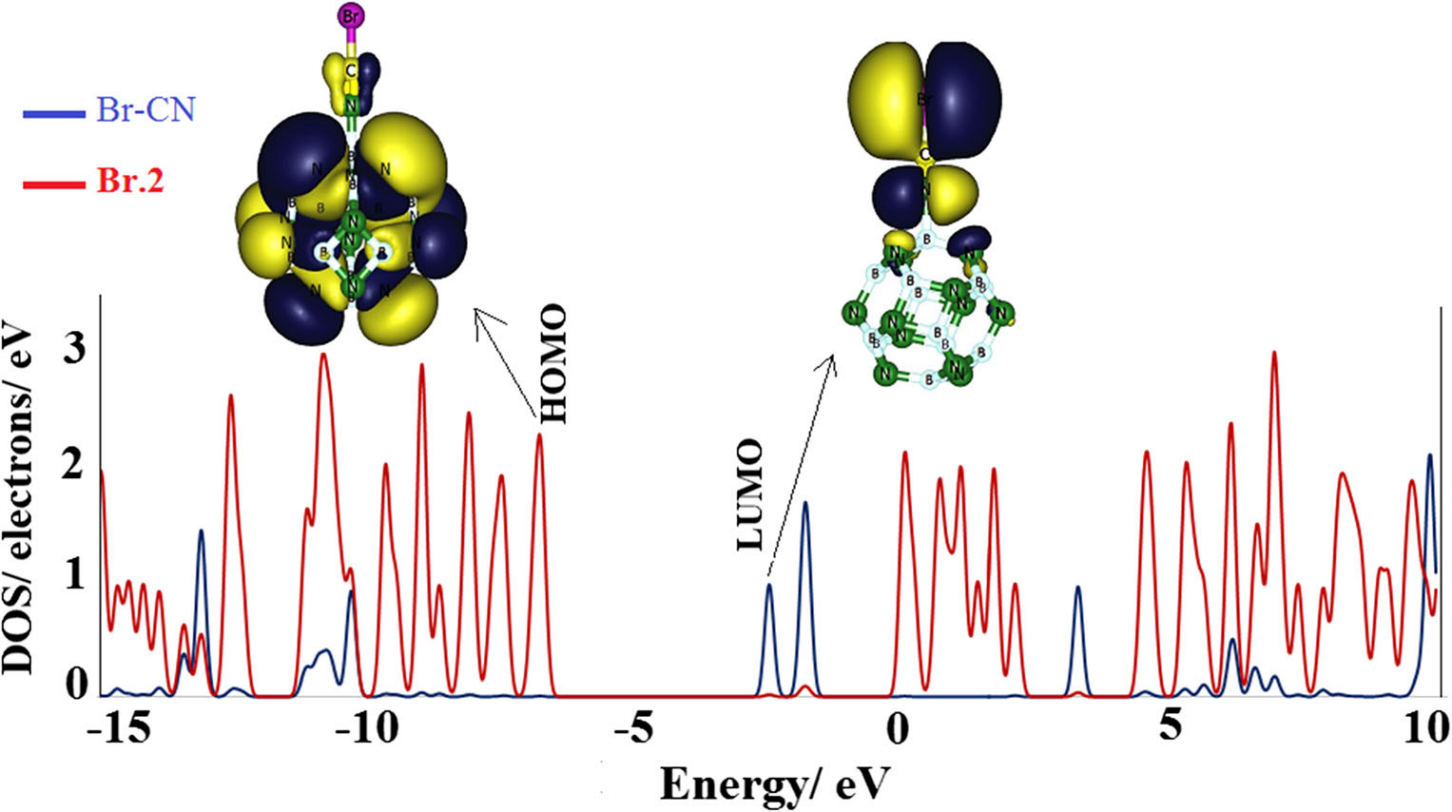
Partial density of states (PDOS) of complex **Br.2**, and its HOMO and LUMO profiles

After charge transfer from the X–CN to the BN cluster, the X–CN molecule becomes partially positive and suitable for LUMO level in the complexes. By increasing the charge transfer, the X–CN becomes more positive and the LUMO level is more stabilized (Table 2). Our partial DOS plot analysis for complex **Br.2** (as a representative model) in Fig. 5 indicates that the newly appeared state is LUMO level, and is created mainly by the contribution of the Br–CN molecule. Frontier molecular orbital analysis shows that, in accordance with the energy change, the LUMO level is shifted from the surface of the BN cage to the surface of Br–CN (Fig. 5). These findings indicate that the presence of X–CN molecules will boost the electrical conductivity of the B_12_N_12_ nanocage, which, by increasing the atomic number of X atoms, increases the electrical conductivity more. It can be concluded that X–CN can be detected selectively by B_12_N_12_ because a different electrical signal will be produced upon adsorption of the B_12_N_12_ nanocluster.

### Recovery time

Sensor recovery from the adsorbed gases is of great importance. Experimentally the recovery process is done by heating to upper temperatures or by UV light exposure [81]. The recovery time can be calculated from transition theory:

5 $$ \tau ={\upupsilon}^{-1} \exp \left(-{\mathrm{E}}_{\mathrm{ad}}/\mathrm{kT}\right) $$

where k is Boltzmann’s constant (∼8.31 × 10^−3^ kJ mol^−1^.K), T is temperature, and υ the attempt frequency. If one employs an attempt frequency of about 10^12^ s^−1^ (which has been used to recover carbon nanotubes at room temperature [82]), the recovery time of Br–CN, Cl–CN, and F–CN molecules in complexes **Br.2**, **Cl.2**, and **F.2** will be about 1.43, 0.15, and 0.02 ms, respectively. This shows that the B_12_N_12_ nanocluster benefits from a short recovery time as a sensor. As a comparison, it has been shown experimentally that the recovery time for NO_2_ desorption from the surface of N-doped carbon nanotubes is about 9 ms, which is excellent [83].

## Conclusions

We investigated the adsorption of X–CN molecules on the BN nanocage using DFT calculations. We found that this cluster may be a promising gas sensor for detection of X–CN gases because of a large charge transfer and the reduction of* E*_g_ of the cage. It was shown that the cage can selectively detect these gases because of their different effect on the electrical conductivity. Increasing the atomic number of the X atom, the LUMO level is much more stabilized, and the* E*_g_ is much more reduced. The X–CN molecules prefer to be adsorbed on the B sites of the BN cluster via their N-head, with Δ*H*_ad_ values of about −19.2 kJ mol^−1^, −23.4 kJ mol^−1^, and −30.5 kJ mol^−1^ for X = F, Cl, and Br, respectively. Also, the recovery time of the Br–CN, Cl–CN, and F–CN molecules in complexes **Br.2**, **Cl.2**, and **F.2** was calculated to be 1.43 ms, 0.15 ms, and 0.02 ms, respectively.
